# A meta-analysis of narrow band imaging for the diagnosis and therapeutic outcome of non-muscle invasive bladder cancer

**DOI:** 10.1371/journal.pone.0170819

**Published:** 2017-02-13

**Authors:** YiQuan Xiong, JianDong Li, ShuJuan Ma, Jing Ge, LiZhi Zhou, Dongliang Li, Qing Chen

**Affiliations:** 1 Department of Epidemiology, School of Public Health and Tropical Medicine, Southern Medical University, Guangdong Provincial Key Laboratory of Tropical Disease Research, Guangzhou, China; 2 School of Public Health, Central South University, Changsha, Hunan, China; 3 Department of Biostatistics, School of Public Health and Tropical Medicine, Southern Medical University, Guangzhou, China; University of Oklahoma Health Sciences Center, UNITED STATES

## Abstract

**Objectives:**

To assess the additional detection rate (ADR) of within-patient comparisons of Narrow band imaging (NBI) and white light cystoscopy (WLC) for non-muscle invasive bladder cancer (NMIBC) detection and compare the impact of NBI and WLC on bladder cancer recurrence risk.

**Methods:**

We searched relevant studies from PubMed, Embase, Medline, Web of Science and the Cochrane Library database for all articles in English published beforeJuly26^th^, 2016. Pooled ADR, diagnostic accuracy, relative risk (RR) and their 95% confidence intervals (CIs) were calculated.

**Results:**

Twenty-five studies including 17 full texts and eight meeting abstracts were included for analysis. Compared to WLC, pooled ADR of NBI for NMIBC diagnosis was 9.9% (95% CI: 0.05–0.14) and 18.6% (95% CI: 0.15–0.25) in per-patient and per-lesion analysis, respectively. Pooled ADR of NBI for carcinoma in situ (CIS) diagnosis was 25.1% (95% CI: 0.09–0.42) and 31.1% (95% CI: 0.24–0.39) for per-patient and per-lesion analyses, respectively. The pooled sensitivity of NBI was significantly higher than WLC both at the per-patient (95.8% vs. 81.6%) and per-lesion levels (94.8% vs. 72.4%). In addition, NBI significantly reduced the recurrence rate of bladder cancer with a pooled RR value of 0.43 (95% CI: 0.23–0.79) and0.81 (95% CI: 0.69–0.95) at month three and twelve, respectively.

**Conclusions:**

NBI is a valid technique that improves the diagnosis of NMIBC and CIS compared to standard WLC either at per-patient or per-lesion level. It can reduce the recurrence rate of bladder cancer accordingly.

## Introduction

Bladder cancer (BC) is the fourth most common malignancy in men and ninth in women [1,2].The incidence of BC is rapidly increasing in underdeveloped countries. Approximately 80% of diagnosed bladder tumors are non-muscle invasive bladder cancer (NMIBC) [3].White light cystoscopy (WLC) is the standard imaging tool to identify suspicious lesions, detect cancer and tumor recurrence in bladder. Once a lesion is identified, transurethral resection (TUR), the mainstay of treatment for NMIBC, will be performed to assess histopathologic grade and stage. Despite its central role, WLC has several well recognized limitations. It is difficult to visualize non-papillary bladder cancer using WLC, such as carcinoma in situ (CIS), and small, or satellite tumors[4].In addition, bladder cancer may be incompletely resected because of understaging[5]. These limitations of WLC contribute to the high risk of cancer persistence and high recurrence rate (approximately 61% at year one and 78% at year five) [6,7]. Due to the high prevalence, high recurrence rate, and the need for long-term cystoscopic surveillance, BC has a tremendous impact on healthcare infrastructure and costs[8].

NBI is a valid technique that can improve bladder cancer detection. NBI filters out the red spectrum of white light, resulting in blue (415 nm) and green (540 nm) bands that can differentially penetrate mucosa to enhance visualization of mucosal vasculature and highlight neoplastic neoangiogenesis of urothelial tumors. There is a commercially available NBI system (Olympus Corp, Tokyo, Japan) used to detect BC. Urologists can change the optical setting on these devices to toggle between WLC and NBI [9].To date, an increasing number of studies, which focused on evaluating the additional detection rate (ADR) of NBI for BC compared with WLC, have been published with a variety of findings. However, there was only one meta-analysis included seven studies (data search up to April 2012)compared the detection rate of NMIBC between NBI and WLC [10]. After the previous meta-analysis, many relevant original studies were published. It is necessary to update the pooled ADR of NBI for BC compared with WLC with the latest evidences. Besides, there is still lack of evidence from the direct comparison of NBI and WLC for same patients, and so it is still unclear whether there is any significant advantage in the clinical use of NBI compared with WLC.

To achieve a comprehensive analysis in order to guide rational use of NBI based on the latest evidence, we performed a meta-analysis to assess the ADR of within-patient comparisons of NBI and WLC for NMIBC detection and compare the impact of NBI and WLC on bladder cancer recurrence risk.

## Methods

This meta-analysis was conducted following the guidelines of Preferred Reporting Items for Systematic reviews and Meta-Analyses (PRISMA)[11].

### Literature search

We searched PubMed, Embase, Medline, Web of Science and the Cochrane Library database from the earliest date available through July26^th^, 2016 in English. We used following keywords, separately and in combinations: “bladder tumor”, “bladder cancer”, “urothelial cancer”, “UC”, “non-muscle invasive bladder cancer”, “NMIBC”, “carcinoma in situ”, “CIS”, “narrow band imaging” and “NBI”. Forward citation searching and hand searching of reference lists were also conducted.

### Selection criteria

Studies were included if they met following criteria:(1) they evaluated ADR and/or recurrence rate of NMIBC; (2) they provided effective comparison groups (NBI vs. WLC); (3)they reported sufficient data including detected and total number of NBI and WLC at a per-patient or per-lesion level (a lesion was defined as a biopsy specimen or a biopsy location), or total number of subjects and recurrence rate of NBI and WLC during the follow-up period of at least three months, or provided sufficient data for their estimation. When there were multiple publications from the same population during an overlapping time period, only the study with the largest series patients was included.

Studies were excluded if: (1) they were reviews, editorials, opinions, animal models or case reports; (2)they only evaluated the ADR and/orrecurrence rate of NBI or WLC for NMIBC; (3) no sufficient data of ADR and/or recurrence rate could be extracted; (4) patients were undergoing the procedure without pathological confirmation of lesions.

### Data selection and extraction

Citations were merged in EndNote version X7 (Thomson Reuters) to facilitate management. Two authors (Li JD and Ma SJ) evaluated all retrieved articles by title and abstract in an unblinded standardized manner, to determine whether a paper met the inclusion criteria. Studies that fulfilled the inclusion criteria after full-text screening were finally included in quantitative synthesis. We extracted relevant data from each eligible study for first author, study year, country of origin, study setting, number of enrolled patients or lesions, sex ratio, detected and total number of NBI and WLC, recurrence, and total number of patients in NBI-TUR and WLC-TUR groups. Data for per-patient and per-lesion analyses were extracted separately whenever available. Data extraction was by two authors (Li JD and Ma SJ) independently and consensus was reached on all items.

### Quality assessment

The quality and risk of bias of included diagnostic studies were assessed using Quality Assessment of Diagnostic Accuracy Studies-2 (QUADAS-2)[12]. The QUADAS-2 tool consists of four key domains including patient selection, index test, reference standard, flow and timing. Risk of bias was judged as “low”, “high”, or “unclear”.

### Statistical analysis

ADR was defined as the additional number (patients or lesions) of NBI detected divided by the total number (patients or lesions) of NBI and WLC detected. The between-study heterogeneity was estimated using the *I*^*2*^ statistic. Significant heterogeneity was defined as *I*^*2*^ exceeding 50%. Pooled results of ADR, relative risk (RR), sensitivity, specificity, positive likelihood ratio (PLR), negative likelihood ratio (NLR) and corresponding 95% confidence intervals (CIs) were calculated using the fixed effects model (Mantel and Haenszelmethod) when heterogeneity was not significant (*I*^*2*^<50%). Otherwise, a random-effects model was applied. Forest plots were constructed for visual display of pooled results if necessary. Publication bias was examined using Egger’s linear regression text and “trim and fill” method. Meta-regression was applied to detect the potentially important covariates exerting substantial impact on between-study heterogeneity. Statistical analyses were conducted using Meta-Disc software (version 1.4; Unit of Clinical Biostatistics, Ramony Cajal Hospital, Madrid, Spain) [13] and STATA 12.0 (Stata Corp LP, College Station, TX, USA).

## Results

### Description of included studies

**Fig 1:**
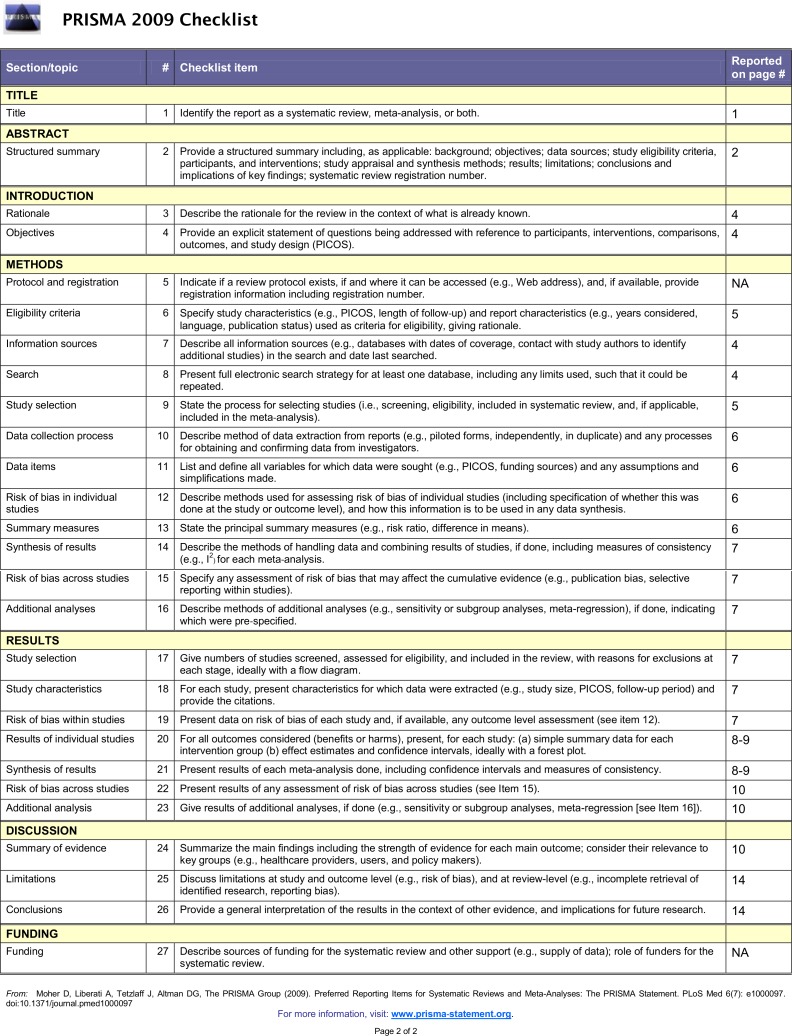
The PRISMA checklist for this meta-analysis.

Of 856 potentially relevant studies generated by the literature search, 25 studies [14–38]including 17 full texts and eight meeting abstracts were eligible for analysis. The selection process is shown in S1 Fig. The PRISMA checklist was showed in Fig 1. Twenty studies[14–33], covering a total of 2,806 patients, reported the ADR results of within-patient comparisons of NBI and WLC for NMIBC detection. The main characteristics of these studies are described in Table 1. Six studies[16,34–38], covering a total of 1,557 patients, reported the recurrence rates using NBI compared with WLC. The main characteristics of these six studies are described in Table 2. Eight studies [14–16,18,21,24,29,33]reported the diagnostic accuracy of NBI and WLC in detection of NMIBC.

**Table 1:** Basic characteristic of the eligible studies for additional detection rate analysis.

Author	Year	Country	Study style	Study design	Center(n)	Patients(n)	Age(mean or median)	Male (%)
Ye^14^	2015	China	full text	prospective	eight	103	62	78.4
Herr^15^	2008	America	full text	prospective	one	427	65	74
Kohei^16^	2015	Japan	full text	retrospective	one	57	75	84.2
Chen^17^	2013	China	full text	prospective	one	179	53.6	61.5
Katsunori^18^	2010	Japan	full text	prospective	four	104	70.6	84.6
Cauberg^19^	2010	Netherlands/Czech Republic	full text	prospective	two	95	70.6	73.7
Shadpour^20^	2016	Iran	full text	prospective	one	50	63.8	68
Shen^21^	2012	China	full text	prospective	one	78	68	79.5
Bryan^22^	2008	United Kingdom	full text	prospective	one	29	NA	NA
Zhu^23^	2011	China	full text	retrospective	one	12	57	75
Song^24^	2016	Korea	full text	prospective	one	63	66	61.9
Jecu^25^	2014	Romania	full text	retrospective	one	253	NA	70
Bryan^26^	2010	United Kingdom	full text	prospective	one	23	NA	NA
Naselli^27^	2009	Italy	full text	prospective	one	47	62	83
Giulianelli^28^	2015	Italy	abstract	NA	one	797	NA	NA
Dalgaard^29^	2015	Denmark/Norway/Spain/France	abstract	prospective	four	68	NA	NA
Lam^30^	2013	United Kingdom	abstract	prospective	one	152	NA	NA
Saltirov^31^	2011	Bulgaria	abstract	NA	one	64	NA	NA
Jensen^32^	2012	Denmark	abstract	NA	one	52	NA	NA
Drejer^33^	2016	Denmark	abstract	NA	three	153	NA	NA

Note: NA, no data available.

**Table 2:** Basic characteristic of the eligible studies for recurrencerate analysis.

Author	Year	Country	Study style	Center(n)	Total patients(n)	NBI			WLC			Followed time (month)
						Patients(n)	Age(mean or median)	Male (%)	Patients(n)	Age(mean or median)	Male (%)	
Naselli^34^	2012	Italy	full text	two	148	76	70.8	15.8	72	71.6	23.6	3 and 12
Kohei^16^	2015	Japan	full text	one	135	57	75	84.2	78	73	79.5	3 and 12
Cauberg^35^	2011	Netherlands	full text	one	158	40	67.9	75	120	67.8	71.7	3
Montanari^37^	2012	Italy	full text	one	92	47	NA	NA	45	NA	NA	12
Lee^36^	2014	Korea	abstract	one	68	33	63.8	NA	35	63.03	NA	10 and 25
Naito^38^	2015	Japan	abstract	Multicenter	956	484	NA	NA	481	NA	NA	12

Note: RCT, randomized controlled trial; NBI,narrow band imaging;WLC,white light cystoscopy; NA, no data available.

### Quality assessment

Results of the assessment of study quality are shown in S1 Table. In three studies[22,26,28], risk of bias in patient selection was unclear, as unable to determine whether the patients in these studies were continuously enrolled or not. One study[24] had a high risk of bias in the patient selection as included patients had confirmed NMIBC before endoscopy. As for the index test item of NBI in applicability concerns, three studies[15,16,32] scored a high risk of bias, in which NBI was followed by WLC to identify positive lesions and investigate whether any additional bladder was available.

### Analysis of additional detection rate

**Fig 2:**
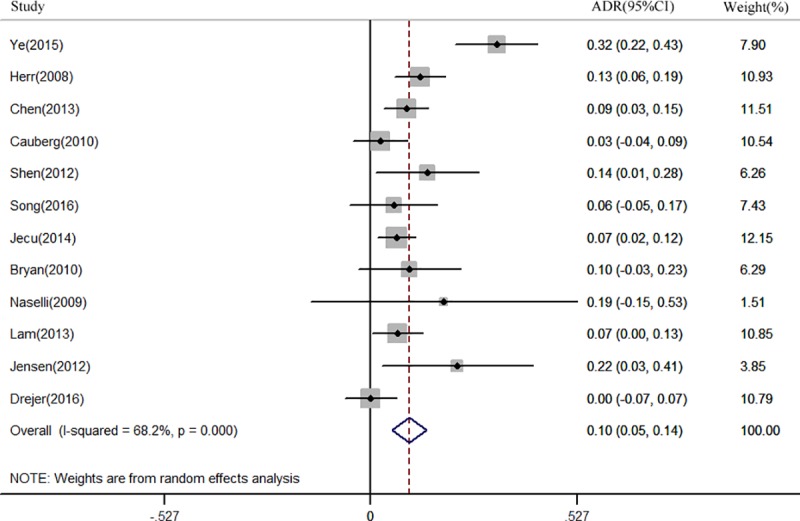
Forest plot of the pooled additional detection rate (ADR) of Narrow-band imaging (NBI) when compared to White light cystoscopy (WLC) for non-muscle invasive bladder cancer (NMIBC) detection in per-patient analysis.

**Fig 3:**
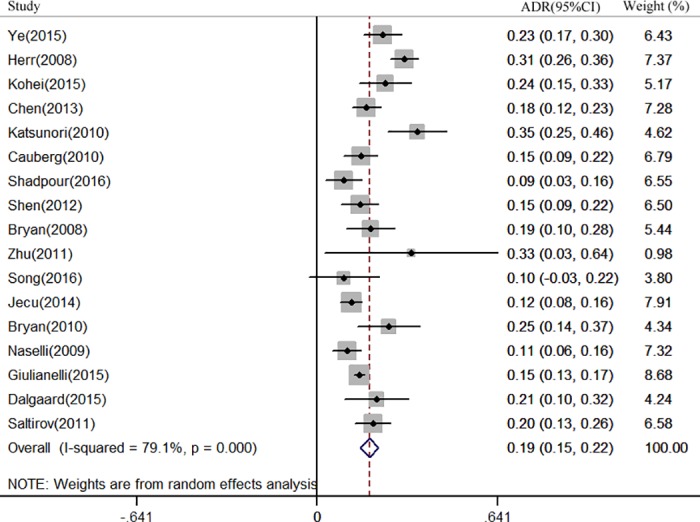
Forest plot of the pooled additional detection rate (ADR) of Narrow-band imaging (NBI)when compared toWhite light cystoscopy (WLC)for non-muscle invasive bladder cancer (NMIBC) detection in per-lesion analysis.

Twelve studies [14,15,17,19,21,24–27,30,32,33] involving 1,625 patients reported a per-patient analysis of NBI for NMIBC detection. The ADR ranged from 0 to 32%. The pooled result for ADR was 9.9% (95% CI: 0.05–0.14, *I*^2^ = 68.2%) (Fig 2). Correspondingly, Seventeen studies [14–29,31] were included for per-lesion analysis. The ADR ranged from 9% to 35%.Pooled ADR was 18.6% (95% CI: 0.15–0.25, *I*^2^ = 79.1%) (Fig 3).When only considering the prospective studies, nine studies [14,15,17,19,21,24,26,27,30]involving1167 patients were included for per-patient analysis and the pooled ADR was 11.3% (95% CI: 0.06–0.17, *I*^2^ = 67.4%). Twelve studies[14,15,17–22,24,26,27,29] were included for per-lesion analysis and the pooled ADR was 19.2% (95% CI: 0.15–0.24, *I*^2^ = 81.2%).

**Fig 4:**
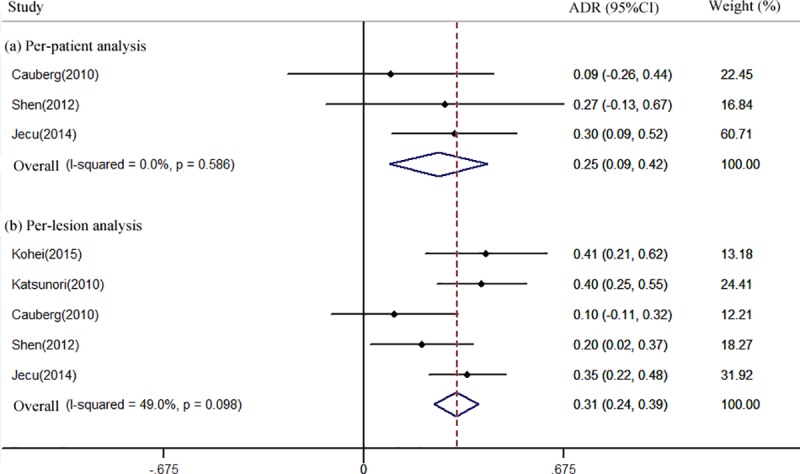
Forest plot of the pooled additional detection rate (ADR) of Narrow-band imaging (NBI)when compared toWhite light cystoscopy (WLC)for carcinoma in situ (CIS) detection in per-patient (a) and per-lesion analysis (b).

In addition, three studies[19,21,25] involving 45 patients reported per-patient analysis of NBI for CIS detection. The ADR ranged from 9% to 30%.Corresponding pooled results for ADR was 25.1% (95% CI: 0.09–0.42, *I*^2^ = 0.0%) (Fig 4A). Five studies [16,18,19,21,25] involving 225 lesions reported per-lesion analysis of NBI for CIS detection. The ADR ranged from 10% to 41%.Pooled ADR was 31.1% (95% CI: 0.24–0.39, *I*^2^ = 49.0%) (Fig 4B).

### Diagnostic accuracy

Five studies [14,15,21,24,33] involving824 patients reported the diagnostic accuracy of NBI and WLC in detection of NMIBC per-patient. Six studies [14,16,18,21,24,29] involving 1518 lesions reported diagnostic accuracy per-lesion. In per-patient analysis, the pooled sensitivity and specificity of NBI were 95.8% (95% CI: 0.93–0.98, *I*^2^ = 80.1%) and 73.6% (95% CI: 0.69–0.78, *I*^2^ = 89.4%), respectively (Table 3). Pooled sensitivity and specificity of WLC were 81.6% (95% CI: 0.77–0.85, *I*^2^ = 87.7%) and 79.2% (95% CI: 0.75–0.83, *I*^2^ = 92.2%), respectively (Table 3).

**Table 3:** Diagnostic accuracy of NBI and WLC in detection of NMIBC.

analysis	Number of studies (patient or lesions)	Sensitivity(95% CI)	Heterogeneity (*I*^2^)	Specificity (95% CI)	Heterogeneity (*I*^2^)	Positive LR(95% CI)	Heterogeneity (*I*^2^)	Negative LR (95% CI)	Heterogeneity (*I*^2^)	AUC
**Per-patient**										
NBI	5 (824)	95.8% (0.93–0.98)	80.1%	73.6% (0.69–0.78)	89.4%	2.74 (1.62–4.63)	90.7%	0.06 (0.01–0.25)	82.0%	0.849
WLC	5 (824)	81.6% (0.77–0.85)	87.7%	79.2% (0.75–0.83)	92.2%	3.01(1.27–7.14)	95.8%	0.23 (0.09–0.61)	86.6%	0.889
**Per-lesion**										
NBI	6 (1518)	94.8% (0.93–0.96)	61.5%	65.6% (0.62–0.69)	94.9%	2.40 (1.42–4.05)	97.4%	0.09 (0.05–0.15)	52.9%	0.940
WLC	6 (1518)	72.4% (0.69–0.76)	75.1%	79.1% (0.76–0.82)	93.2%	3.15 (1.99–4.99)	90.7%	0.37 (0.29–0.48)	68.1%	0.812

Note: AUC, area under the curve; CI, confidence interval.

In per-lesion analysis, pooled sensitivity and specificity of NBI were 94.8% (95% CI: 0.93–0.96, *I*^2^ = 61.5%) and 65.6% (95% CI: 0.62–0.69, *I*^2^ = 94.9%), respectively (Table 3). Pooled sensitivity and specificity of WLC were 72.4% (95% CI: 0.69–0.76, *I*^2^ = 75.1%) and 79.1% (95% CI: 0.76–0.82, *I*^2^ = 93.2%), respectively (Table 3).

### Recurrence rate

**Fig 5:**
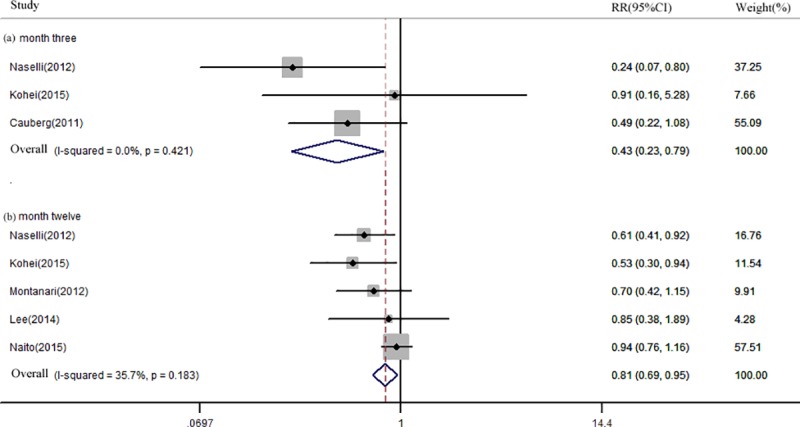
Forest plot of the pooled relative risk (RR) for Narrow-band imaging (NBI) compared to White light cystoscopy (WLC) at month three (a) and twelve (b).

Six studies[16,34–38]involving1,557 patients reported recurrences rate of NMIBC. Three studies[16,34,35], five studies[16,34,36–38],and one studies[36],reported recurrence rates of NBI and WLC at month 3, 12, and 24, respectively. The pooled recurrence rates of the NBI group at month 3 and 12 were 4.6% (95% CI: 0.02–0.08, *I*^2^ = 45.6%) and 26.0% (95% CI: 0.23–0.29, *I*^2^ = 0.0%). Correspondingly, pooled recurrence rates of the WLC group at month 3 and 12 were 16.7% (95% CI: 0.003–0.33, *I*^2^ = 94.0%) and 38.6% (95% CI: 0.28–0.50, *I*^2^ = 83.4%), respectively. The pooled RR for NBI when compared to WLC at month 3 and 12 were 0.43 (95% CI: 0.23–0.79, *I*^2^ = 0.0%) and 0.81 (95% CI: 0.69–0.95, *I*^2^ = 35.7%) (Fig 5).

### Heterogeneity analysis

Between-studies heterogeneity of ADR in per-patient and per-lesion analysis was explored by meta-regression. We included four variables: (1) study design (prospective or retrospective); (2) study type (full text or abstract); (3) study center (single or multiple) and (4) number of patients (<50or ≥50) or lesions (<100 or ≥100).The meta-regression analysis did not reveal any factor that contributed to the heterogeneity.

### Publication bias

Egger’s linear regression text showed no statistically significant publication bias of ADR in per-patient analysis (*p* = 0.16) and per-lesion analysis (*p* = 0.11) (S2 Fig). Publication bias in per-lesion analysis (*p* = 0.40) of NBI for CIS detectionwere also not significant. Deek’s test showed no statistically significant publication bias in diagnostic accuracy of NBI (*p* = 0.11) and WLC (*p* = 0.85) in per-lesion analysis.

## Discussion

This meta-analysis synthesized published evidence about the ADR of within-patient comparisons of NBI and WLC for NMIBC diagnosis and therapeutic outcome. Our results indicated that NBI increased NMIBC detection by 9.9% at the per-patient level and 18.6% at the per-lesion level. The pooled sensitivity of NBI was significantly higher than WLC both at the per-patient (95.8% vs. 81.6%) and per-lesion levels (94.8% vs. 72.4%). In addition, NBI significantly reduced the recurrence rate of BC after TUR with a pooled RR value of 0.43 and 0.81 at month 3 and 12, respectively.

Compared with the previous meta-analysis conducted by Li *et al*.[10], the pooled ADRs of NBI for NMIBC diagnosis in our study were lower both at per-patient level [17% (95% CI:0.10–0.25) vs. 9.9% (95% CI: 0.05–0.14)] and per-lesion level [24% (95% CI:0.17–0.31) vs. 18.6% (95% CI: 0.15–0.25)].Although the pooled ADRs of NBI for NMIBC diagnosis in our study were lower than the previous meta-analysis[10], the results in our meta-analysis may more moderate and that could reflected the value of NBI in clinical practice as more original studies and patients were included in our study.

Because of the higher sensitivity of NBI, more tumors can be detected. Herr*et al*.[15] reported a higher number of identified tumors per patient visualized on NBI cystoscopy (3.4) than WLC cystoscopy (2.3). Similar results were shown in a study conducted by Bryan *et al*.[26], which indicated that NBI identified 2.6 tumors per patient while WLC identified only 1.9. However, several other studies showed that the number of tumors identified per patient by NBI and WLC was similar[21,22,39].

Photodynamic detection (PDD) is another new diagnostic and imaging tool for establishing a diagnosis of bladder cancer. Although sufficient evidence indicates that when compared to WLC, both PDD and NBI can improve diagnosis and reduce the recurrence rate of bladder cancer[10,40–42],few trials directly compare the two techniques. A preliminary study conducted by Yoshio *et al*.[43]firstly reported the comparison of PDD and NBI in the same patients with flat urothelial lesions suspicious of CIS of the bladder. The results indicated that the sensitivity and specificity of PDD and NBI were similar (91.6% vs 62.5 and 82.7% vs. 87.9%).A network meta-analysis[44], assessing the therapeutic outcome of TUR in patients with NMIBC assisted by PDD or NBI, showed that resection using NBI and PDD did not differ significantly in terms of cancer recurrence rate [Hexaminolevulinic acid (HAL)-based PDD vs. NBI, OR = 1.11, 95% CI (0.55–2.1); 5-aminolevulinic acid (5-ALA)-based PDD vs. NBI, OR = 0.53, 95% CI (0.26–1.09)].Lacking enough direct evidence, there could not determine whether the performance of PDD and NBI in BC diagnosis were similar or not.

Higher sensitivity may bring a higher false-positive rate (FPR) and result in additional biopsies. The reported FPR of NBI ranged from 21.8% to 50% at per-patient level[14,15,39] and ranged from 13.6% to 39.1% at per-lesion level.[14,18,19,25,45,46] Cauberg*et al*.[19] found FPR of NBI was significantly higher than WLC (31.6% vs. 24.5%, *p*<0.01) at per-lesion level. Additionally, a similar result was found by Katsunori*et al*.[18] (29.1% vs. 13.8%, *p*<0.01). A previous meta-analysis [10] including four studies showed a slightly higher false positive detection rate (FDR) of NBI than WLC in tumor level without significant difference. Our results showed the specificity of NBI was significantly lower than WLC at per-lesion level in within-patient comparisons. However, another meta-analysis [47] showed no significant specificity difference between NBI and WLC (84.7% vs. 87.0%). Whether the FDR and specificity of NBI are indeed different to WLC needs to be further explored.

Although NBI provides a subjective impression of abnormal areas of bladder mucosa without the use of dyes, it does not appear to have a significant associated learning curve.[26,48] Bryan *et al*.[26] reported that a new NBI user demonstrated a significantly improved detection rate of urothelial carcinoma (UC) with NBI compared to WLC with an ADR of 35%. When compared to an experienced user, there was no significant difference in the excess number of detected UC (*p* = 0.74). Herr *et al*.[48] also found no significant difference between new and experienced users of NBI in the detection rate of recurrent bladder tumors.

Several studies showed that NBI significantly reduced the recurrence rate of BC.[15,16,19,39] Kohei*et al*.[16] reported that the recurrence rate at month 12 in the NBI-TUR group was significantly lower than in the WLC-TUR group (21.1% vs. 39.7%, *p* = 0.016). A meta-analysis [44]including four studies showed that NBI-TUR was superior to WLC-TUR, with an RR of 0.47 (95% CI: 0.31–0.72). However, in this meta-analysis, the author did not show the short-term or long-term impact of NBI-TUR on the recurrence rate of BC. In our results, the pooled recurrence rates of NBI and WLC at month 3 and 12 were 4.6% vs. 16.7% and 26.0% vs. 38.6%, respectively. Pooled RRs for NBI at month 3 and 12 were 0.43 (95% CI: 0.23–0.79) and 0.81 (95% CI: 0.47–0.77), respectively. Limited by the small number of patients in included studies, the value of NBI as an aid to TUR in reducing the long-term recurrence rate needs further evaluation in randomized controlled trials.

Even though several studies have shown that NBI objectively improves the detection of primary and recurrent BC, there are still some controversies. One controversial area is potential observer bias. In instances where WLC and NBI were performed subsequently by the same urologist, the increased detection rate by NBI may result from the ‘‘second look” inspection of the bladder. Herr*et al*.[15] reported that subtle tumors recognized first with NBI also became visible when the image was switched back to WLC in several cases. In order to address observer bias, Shen *et al*.[21] performed NBI and WLC to detect BC in a randomized imaging sequence modality. According to the randomization protocol, the bladder was mapped using WLC then NBI, or vice versa during the same observation period. The result showedthat NBI still identified significantly more additional tumors than WLC, confirming that a “second look” did not compromise the superiority of NBI over standard WLC for detecting primary NMIBC including CIS lesions.

This meta-analysis had four limitations. First, there was significant heterogeneity for some major results. Different inclusion and exclusion criteria and observer experience bias might contribute to such heterogeneity. Although we used a random-effects model, there was still some influence on the final results. Second, quality assessment showed that not all the included studies were high quality, as some indices were labeled as high risk bias diagnostic studies, which might lead to some bias in the final statistical results. Third, studies included to evaluate the diagnostic accuracy of NBI may be uncomprehensive. Since the main objective of this study was to perform an analysis of within-patient comparisons of NBI and WLC, studies that only reported the performance of NBI were excluded. Fourth, the limited number of patients and lesions of CIS in the pooled analysis mean that the results of NBI for CIS detection should be interpreted with caution.

## Conclusions

In conclusion, our meta-analysis indicated that NBI improved the diagnosis of NMIBC and CIS compared to standard WLC either at the per-patient or per-lesion level. This diagnostic test could reduce the recurrence rate of BC accordingly.

## Supporting information

S1 Fig Flow diagram of the studies identified in the meta-analysis. (DOC)

S2 Fig Results of Egger’s linear regression text showing no statistically significant publication bias in per-patient analysis (a) (*P* = 0.16) and per-lesion analysis (b) (*P* = 0.11) of additional detection rate (ADR) in non-muscle invasive bladder cancer (NMIBC) detection.
(TIF)

S1 Table QUADAS-2 Risk of bias assessment. (DOCX)

## References

[1] Siegel R, Naishadham D, Jemal A (2012) Cancer statistics, 2012. CA Cancer J Clin 62: 10–29. 10.3322/caac.20138 22237781

[2] van Lingen AV, Witjes JA (2013) Current intravesical therapy for non-muscle invasive bladder cancer. Expert Opin Biol Ther 13: 1371–1385. 10.1517/14712598.2013.824421 23957696

[3] Babjuk M, Oosterlinck W, Sylvester R, Kaasinen E, Bohle A, Palou-Redorta J, et al (2008) EAU guidelines onnon-muscle-invasive urothelial carcinoma of the bladder. Eur Urol 54: 303–314. 10.1016/j.eururo.2008.04.051 18468779

[4] Fradet Y, Grossman HB, Gomella L, Lerner S, Cookson M, Albala D, et al (2007) A comparison ofhexaminolevulinate fluorescence cystoscopy and white light cystoscopy for the detection ofcarcinoma in situ in patients with bladder cancer: a phase III, multicenter study. J Urol 178: 68–73;discussion 73. 10.1016/j.juro.2007.03.028 17499291

[5] Zlatev DV, Altobelli E, Liao JC (2015) Advances in imaging technologies in the evaluation of high-grade bladder cancer. Urol Clin North Am 42: 147–vii. 10.1016/j.ucl.2015.01.001 25882557 PMC4402158

[6] Morgan TM, Keegan KA, Clark PE (2011) Bladder cancer. Curr Opin Oncol 23: 275–282. 10.1097/CCO.0b013e3283446a11 21311329

[7] Sylvester RJ, van der Meijden AP, Oosterlinck W, Witjes JA, Bouffioux C, Denis L, et al (2006)Predicting recurrence and progression in individual patients with stage Ta T1 bladder cancer using EORTC risk tables: a combined analysis of 2596 patients from seven EORTC trials. Eur Urol 49: 466–465; discussion 475–467. 10.1016/j.eururo.2005.12.031 16442208

[8] Sievert KD, Amend B, Nagele U, Schilling D, Bedke J, Horstmann M, et al (2009) Economic aspects of bladder cancer: what are the benefits and costs? World J Urol 27: 295–300. 10.1007/s00345-009-0395-z 19271220 PMC2694315

[9] Altobelli E, Zlatev DV, Liao JC (2015) Role of narrow band imaging in management of urothelial carcinoma. Curr Urol Rep 16. 10.1007/s11934-015-0527-5 26093973

[10] Li K, Lin T, Fan X, Duan Y, Huang J (2013) Diagnosis of narrow-band imaging in non-muscle-invasive bladder cancer: a systematic review and meta-analysis. Int J Urol 20: 602–609. 10.1111/j.1442-2042.2012.03211.x 23113702

[11] Moher D, Liberati A, Tetzlaff J, Altman DG, Group P (2009) Preferred reporting items for systematic reviews and meta-analyses: the PRISMA statement. J Clin Epidemiol 62: 1006–1012. 10.1016/j.jclinepi.2009.06.005 19631508

[12] Whiting PF, Rutjes AW, Westwood ME, Mallett S, Deeks JJ, Reitsma JB, et al (2011) QUADAS-2: a revised tool for the quality assessment of diagnostic accuracy studies. Ann Intern Med 155: 529–536. 10.7326/0003-4819-155-8-201110180-00009 22007046

[13] Zamora J, Abraira V, Muriel A, Khan K, Coomarasamy A (2006) Meta-DiSc: a software for meta-analysis of test accuracy data. BMC Med Res Methodol 6: 31 10.1186/1471-2288-6-31 16836745 PMC1552081

[14] Ye Z, Hu J, Song X, Li F, Zhao X, Chen S, et al (2015) A comparison of NBI and WLI cystoscopy in detecting non-muscle-invasive bladder cancer: A prospective, randomized and multi-center study. Scientific Reports 5. 10.1038/srep10905 PMC4456941 26046790

[15] Herr HW, Donat SM (2008) A comparison of white-light cystoscopy and narrow-band imaging cystoscopy to detect bladder tumour recurrences. BJU International 102: 1111–1114. 10.1111/j.1464-410X.2008.07846.x 18778359

[16] Kobatake K, Mita K, Ohara S, Kato M (2015) Advantage of transurethral resection with narrow band imaging for non-muscle invasive bladder cancer. Oncology Letters 10: 1097–1102. 10.3892/ol.2015.3280 26622632 PMC4509371

[17] Chen G, Wang B, Li H, Ma X, Shi T, Zhang X. (2013) Applying narrow-band imaging in complement with white-light imaging cystoscopy in the detection of urothelial carcinoma of the bladder. Urologic Oncology: Seminars and Original Investigations 31: 475–479. 10.1016/j.urolonc.2011.02.009 22079940

[18] Tatsugami K, Kuroiwa K, Kamoto T, Nishiyama H, Watanabe J, Ishikawa S, et al (2010) Evaluation of narrow-band imaging as a complementary method for the detection of bladder cancer. Journal of Endourology 24: 1807–1811. 10.1089/end.2010.0055 20707727

[19] Cauberg ECC, Kloen S, Visser M, De La Rosette JJMCH, Babjuk M, Soukup V, et al (2010) Narrow band imaging cystoscopy improves the detection of nonmuscle-invasive bladder cancer. Urology 76: 658–663. 10.1016/j.urology.2009.11.075 20223505

[20] Shadpour P, Emami M, Haghdani S (2016) A Comparison of the Progression and Recurrence Risk Index in Non-Muscle-Invasive Bladder Tumors Detected by Narrow-Band Imaging Versus White Light Cystoscopy, Based on the EORTC Scoring System. Nephrourol Mon 8: e33240 10.5812/numonthly.33240 26981499 PMC4779587

[21] Shen YJ, Zhu YP, Ye DW, Yao XD, Zhang SL, Dai B, et al (2012) Narrow-band imaging flexible cystoscopy in the detection of primary non-muscle invasive bladder cancer: a "second look" matters? International Urology and Nephrology 44: 451–457. 10.1007/s11255-011-0036-5 21792663

[22] Bryan RT, Billingham LJ, Wallace DMA (2007) Narrow band imaging flexible cystoscopy in the detection of recurrent urothelial cancer of the bladder. Bju International 99: 21–22. 10.1111/j.1464-410X.2007.07317.x 18005206

[23] Zhu YP, Shen YJ, Ye DW, Wang CF, Yao XD, Zhang SL, et al (2012) Narrow-band imaging flexible cystoscopy in the detection of clinically unconfirmed positive urine cytology. Urologia Internationalis 88: 84–87. 10.1159/000333119 22104957

[24] Song PH, Cho S, Ko YH (2016) Decision Based on Narrow Band Imaging Cystoscopy without a Referential Normal Standard Rather Increases Unnecessary Biopsy in Detection of Recurrent Bladder Urothelial Carcinoma Early after Intravesical Instillation. Cancer Res Treat 48: 273–280. 10.4143/crt.2014.190 25761489 PMC4720086

[25] Jecu M, Geavlete B, Multescu R, Stanescu F, Moldoveanu C, Adou L, et al (2014) NBI cystoscopy in routine urological practice—from better vision to improve therapeutic management. Journal of medicine and life 7: 282–286. 25408740 PMC4197490

[26] Bryan RT, Shah ZH, Collins SI, Wallace DMA (2010) Narrow-band imaging flexible cystoscopy: A new user's experience. Journal of Endourology 24: 1339–1343. 10.1089/end.2009.0598 20629569

[27] Naselli A, Introini C, Bertolotto F, Spina B, Puppo P (2010) Narrow band imaging for detecting residual/recurrent cancerous tissue during second transurethral resection of newly diagnosed non-muscle-invasive high-grade bladder cancer. BJU International 105: 208–211. 10.1111/j.1464-410X.2009.08701.x 19549255

[28] Giulianelli R, Gentile BC, Mirabile G, Albanesi L, Attisani F, Mavilla L, et al (2015) Can NBI cytoscopy increase the detection rate of carcinoma in situ? Our experience. European Urology, Supplements 14: e1047–e1047a.

[29] Dalgaard L, Zare R, Palou Redorta J, Gaya J, Roumiguie M, Filleron T, et al (2015) Flexible full HD videoscope with narrow band imaging improves the detection of NMIBC. European Urology, Supplements 14: e840.

[30] Lam W, Ayres B, Fernando A, Perry M (2013) Narrow band imaging improves the detection of new and recurrent bladder cancers and carcinoma in-situ. European Urology, Supplements 12: e593.

[31] Saltirov I, Petkov T, Petkova K (2011) Narrow-Band Imaging (NBI) flexible cystoscopy in the diagnosis of urothelial carcinoma of the bladder. European Urology, Supplements 10: 597.

[32] Jensen JB, Hoyer S (2012) Narrow-band imaging (NBI) in flexible cystoscopy improves diagnosis of bladder pathology in the outpatient clinic. European Urology, Supplements 11: e446–e446a.

[33] Drejer D. BS, Lam G.W., Jensen J.B. (2016) Comparison of white light, photodynamic diagnosis (PDD) and narrow band imaging (NBI) in detection of flat dysplasia and CIS at transurethral resection of the bladder-the DaBlaCa-8 study. Eur Urol Suppl 2016;15(3);e739. 10.1016/j.urology.2016.11.032 27894979

[34] Naselli A, Introini C, Timossi L, Spina B, Fontana V, Pezzi R, et al (2012) A randomized prospective trial to assess the impact of transurethral resection in narrow band imaging modality on non-muscle-invasive bladder cancer recurrence. European Urology 61: 908–913. 10.1016/j.eururo.2012.01.018 22280855

[35] Cauberg ECC, Mamoulakis C, de la Rosette JJMCH, de Reijke TM (2011) Narrow band imaging-assisted transurethral resection for non-muscle invasive bladder cancer significantly reduces residual tumour rate. World Journal of Urology 29: 503–509. 10.1007/s00345-011-0659-2 21350871 PMC3143329

[36] Lee JY, Choi JH, Kim IK, Jung HD, Kang HW, Lee KS, et al (2014) Recurrence rate of transurethral resection of bladder tumor using narrow band imaging: A randomized control trial, pilot study. Journal of Urology 191: e240–e241.

[37] Montanari E, de la Rosette J, Longo F, Del Nero A, Laguna P (2012) Narrow-band imaging (NBI) and white light (WLI) transurethral resection of the bladder in the treatment of non-muscle-invasive bladder cancer. Arch Ital Urol Androl 84: 179–183. 23427740

[38] S Naito FA, Babjuk M, Bryan R, Herr H, Soloway M, Sun Y, Valiquette L, de la Rosette J (2015) The Clinical Research Office of the Endourology Society (CROES) multicentre randomised trial of narrow band imaging-assisted transurethral resection (TURBT) versus conventional white light-assisted TURBT in primary non-muscle-invasive bladder cancer patients: trial protocol and 1-year results. Journal of Endourology 29(S1): P1–A457 27117749 10.1016/j.eururo.2016.03.053

[39] Chen G, Wang B, Li H, Ma X, Shi T, Zhang X. (2013) Applying narrow-band imaging in complement with white-light imaging cystoscopy in the detection of urothelial carcinoma of the bladder. Urol Oncol Semin Ori 31: 475–479. 10.1016/j.urolonc.2011.02.009 22079940

[40] Stenzl A, Burger M, Fradet Y, Mynderse LA, Soloway MS, Witjes JA, et al (2010) Hexaminolevulinate Guided Fluorescence Cystoscopy Reduces Recurrence in Patients With Nonmuscle Invasive Bladder Cancer. Journal of Urology 184: 1907–1913. 10.1016/j.juro.2010.06.148 20850152 PMC4327891

[41] Burger M, Grossman HB, Droller M, Schmidbauer J, Hermann G, Dragoescu O, et al (2013) Photodynamic Diagnosis of Non-muscle-invasive Bladder Cancer with Hexaminolevulinate Cystoscopy: A Meta-analysis of Detection and Recurrence Based on Raw Data. European Urology 64: 846–854. 10.1016/j.eururo.2013.03.059 23602406

[42] Daniltchenko DI, Riedl CR, Sachs MD, Koenig F, Daha KL, Pflueger H, et al (2005) Long-term benefit of 5-aminolevulinic acid fluorescence assisted transurethral resection of superficial bladder cancer: 5-year results of a prospective randomized study. Journal of Urology 174: 2129–2133. 10.1097/01.ju.0000181814.73466.14 16280742

[43] Naya Y, Oishi M, Yamada Y, Ueda T, Fujihara A, Nakanishi H, et al (2015) Initial experience of combined use of photodynamic diagnosis and narrow band imaging for detection of flat urothelial lesion. International Journal of Clinical Oncology 20: 593–597. 10.1007/s10147-014-0748-5 25228479

[44] Lee JY, Cho KS, Kang DH, Jung HD, Kwon JK, Oh CK, et al (2015) A network meta-analysis of therapeutic outcomes after new image technology-assisted transurethral resection for non-muscle invasive bladder cancer: 5-aminolaevulinic acid fluorescence vs hexylaminolevulinate fluorescence vs narrow band imaging. BMC Cancer 15: 566 10.1186/s12885-015-1571-8 26232037 PMC4521364

[45] Geavlete B, Jecu M, Multescu R, Geavlete P (2012) Narrow-band imaging cystoscopy in non-muscle-invasive bladder cancer: A prospective comparison to the standard approach. Therapeutic Advances in Urology 4: 211–217. 10.1177/1756287212454181 23024703 PMC3441134

[46] Geavlete B, Multescu R, Georgescu D, Stanescu F, Jecu M, Geavlete P. (2012) Narrow band imaging cystoscopy and bipolar plasma vaporization for large nonmuscle-invasive bladder tumors—results of a prospective, randomized comparison to the standard approach. Urology 79: 846–851. 10.1016/j.urology.2011.08.081 22342408

[47] Zheng C, Lv Y, Zhong Q, Wang R, Jiang Q (2012) Narrow band imaging diagnosis of bladder cancer: systematic review and meta-analysis. BJU Int 110: E680–687. 10.1111/j.1464-410X.2012.11500.x 22985502

[48] Herr H, Donat M, Dalbagni G, Taylor J (2010) Narrow-band imaging cystoscopy to evaluate bladder tumours—individual surgeon variability. BJU Int 106: 53–55. 10.1111/j.1464-410X.2009.09119.x 20002669 PMC3137239

