# Supplementary material for: A meta-analysis of narrow band imaging for the diagnosis and therapeutic outcome of non-muscle invasive bladder cancer
Source: PLoS One. 2017 Feb 13;12(2):e0170819. doi: 10.1371/journal.pone.0170819 (PMC5305060; doi:10.1371/journal.pone.0170819)
Supplement: S1 Fig — (DOC) [file pone.0170819.s001.doc]

Records identified through database searching and additional reference checking

(n=856)

Records after duplicates removed

(n=453)

Records excluded by screening of

title and abstract (n=341)

Full-text articles assessed for eligibility

(n=112)

Records excluded by screening of

full-text (n=87)

Review (n=13)

Duplicate publication (n=26)

Series patients report (n=1)

No sufficient data information (n=46)

Retracted (n=1)

Studies included in analysis (n=25)

Full text (n=17)

Abstract (n=8)
