# Supplementary figures and images for: A meta-analysis of narrow band imaging for the diagnosis and therapeutic outcome of non-muscle invasive bladder cancer
Source: PLoS One. 2017 Feb 13;12(2):e0170819. doi: 10.1371/journal.pone.0170819 (PMC5305060; doi:10.1371/journal.pone.0170819)

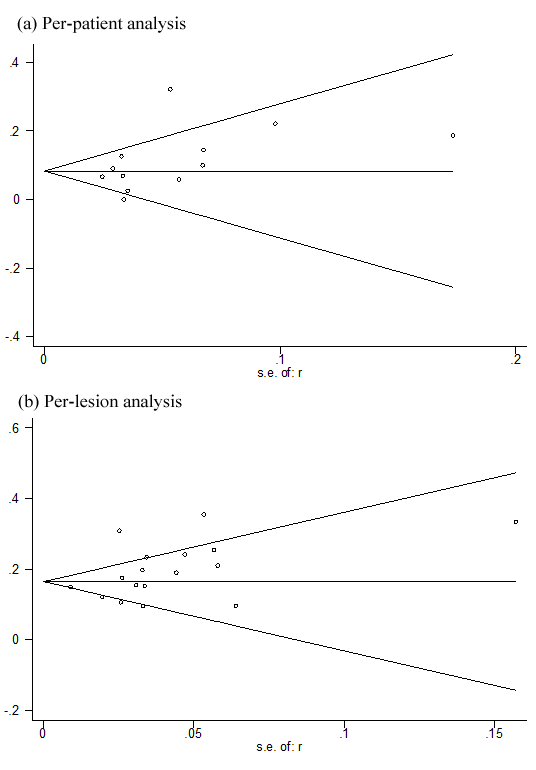

Supplement: S2 Fig — Results of Egger’s linear regression text showing no statistically significant publication bias in per-patient analysis (a) (P = 0.16) and per-lesion analysis (b) (P = 0.11) of additional detection rate (ADR) in non-muscle invasive bladder cancer (NMIBC) detection. (TIF) [file pone.0170819.s002.tif]
