# Supplementary material for: A meta-analysis of narrow band imaging for the diagnosis and therapeutic outcome of non-muscle invasive bladder cancer
Source: PLoS One. 2017 Feb 13;12(2):e0170819. doi: 10.1371/journal.pone.0170819 (PMC5305060; doi:10.1371/journal.pone.0170819)
Supplement: S1 Table — (DOCX) [file pone.0170819.s003.docx]

Supplementary table 1 QUADAS-2 Risk of bias assessment.

| **Study** | **Risk of bias** | | | | |  | **Applicability concerns** | | | |
| --- | --- | --- | --- | --- | --- | --- | --- | --- | --- | --- |
|  | **Patient selection** | **Index test** | | **Reference standard** | **Flow and timing** |  | **Patient selection** | **Index test** | | **Reference standard** |
|  |  | **WLC** | **NBI** |  |  |  |  | **WLC** | **NBI** |  |
| Ye(2015)^14^ | low | low | low | low | low |  | low | low | low | low |
| Herr(2008)^15^ | low | low | low | low | low |  | low | low | High | low |
| Kohei(2015)^16^ | low | low | low | low | low |  | low | low | High | low |
| Chen(2013)^17^ | low | low | low | low | low |  | low | low | low | low |
| Katsunori(2010)^18^ | low | low | low | low | low |  | low | low | low | low |
| Cauberg(2010)^19^ | low | low | low | low | low |  | low | low | low | low |
| Shadpour(2016)^20^ | low | low | low | low | low |  | low | low | low | low |
| Shen(2012)^21^ | low | low | low | low | low |  | low | low | low | low |
| Bryan(2008)^22^ | unclear | low | low | unclear | low |  | low | low | low | low |
| Zhu(2011)^23^ | low | low | low | low | low |  | low | low | low | low |
| Song(2016)^24^ | High | low | low | low | low |  | unclear | low | low | low |
| Jecu(2014)^25^ | low | low | low | low | low |  | low | low | low | low |
| Bryan(2010)^26^ | unclear | low | low | low | low |  | low | low | low | low |
| Naselli(2009)^27^ | low | low | low | low | low |  | low | low | low | low |
| Giulianelli(2015)^28^ | unclear | low | low | low | low |  | low | low | low | low |
| Dalgaard(2015)^29^ | low | low | low | low | low |  | low | low | low | low |
| Lam(2013)^30^ | low | low | low | low | low |  | low | low | low | low |
| Saltirov(2011)^31^ | low | low | low | low | low |  | low | low | low | low |
| Jensen(2012)^32^ | low | low | low | low | low |  | low | low | High | low |
| Drejer(2016)^33^ | low | low | low | low | low |  | low | low | low | low |

Note: low, low risk; high, high risk; unclear, unclear risk. WLC, white light cystoscopy; NBI, narrow-band imaging.
